# {4-Chloro-*N*′-[(2-oxidonaphthalen-1-yl-κ*O*)methyl­idene]benzohydrazidato-κ^2^ *N*′,*O*}di­methyl­tin(IV)

**DOI:** 10.1107/S1600536813031826

**Published:** 2013-11-30

**Authors:** Jichun Cui, Yanling Qiao, Fei Wang

**Affiliations:** a College of Chemistry and Chemical Engineering, Liaocheng University, Shandong 252059, People’s Republic of China

## Abstract

In the title complex, [Sn(CH_3_)_2_(C_18_H_11_ClN_2_O_2_)], the Sn^IV^ ion is coordinated by two O atoms and an N atom from a 4-chloro-*N*′-[(2-oxidonaphthalen-1-yl)methyl­idene]benzohydrazidate ligand and two C atoms from two methyl ligands in a distorted trigonal–bipyramidal geometry [Sn—O = 2.092 (3) and 2.144 (3) Å; Sn—N = 2.160 (4) Å]. The dihedral angle between the naphthalene ring system and the benzene ring is 8.6 (2)°. In the crystal, adjacent mol­ecules are linked by weak C—H⋯O hydrogen bonds, forming a chain along the *b-*axis direction.

## Related literature

For the biological activity and related structures of organotin compounds, see: Hong *et al.* (2013 ▶).
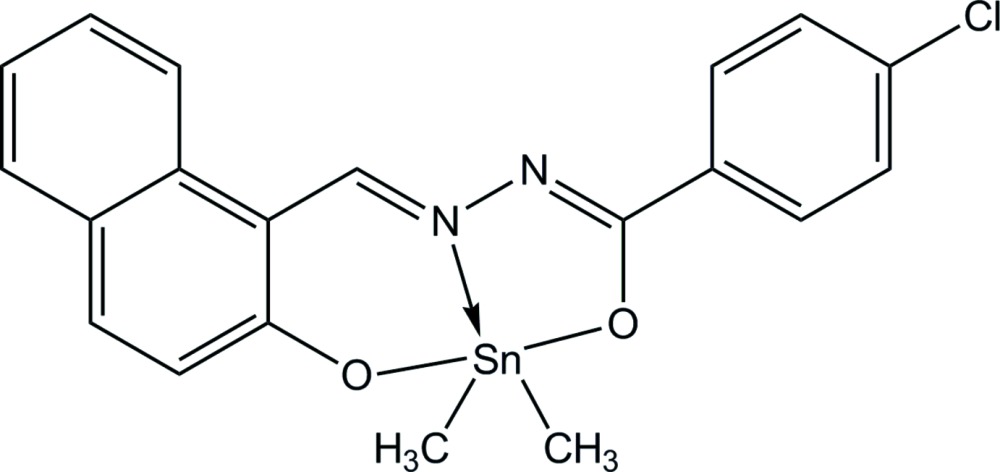


## Experimental

### 

#### Crystal data

[Sn(CH_3_)_2_(C_18_H_11_ClN_2_O_2_)]
*M* *_r_* = 471.50
Monoclinic, 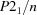

*a* = 8.7927 (8) Å
*b* = 17.4170 (15) Å
*c* = 12.5014 (12) Å
β = 98.690 (9)°
*V* = 1892.5 (3) Å^3^
*Z* = 4
Mo *K*α radiation
μ = 1.51 mm^−1^
*T* = 293 K
0.24 × 0.23 × 0.13 mm

#### Data collection

Siemens SMART CCD area-detector diffractometer
Absorption correction: multi-scan (*SADABS*; Sheldrick, 1996 ▶) *T*_min_ = 0.714, *T*_max_ = 0.828
11127 measured reflections
3343 independent reflections
2584 reflections with *I* > 2σ(*I*)
*R*_int_ = 0.047

#### Refinement

*R*[*F* ^2^ > 2σ(*F* ^2^)] = 0.040
*wR*(*F* ^2^) = 0.107
*S* = 1.06
3343 reflections
237 parameters
H-atom parameters constrained
Δρ_max_ = 0.83 e Å^−3^
Δρ_min_ = −0.61 e Å^−3^

### 

Data collection: *SMART* (Siemens, 1996 ▶); cell refinement: *SAINT* (Siemens, 1996 ▶); data reduction: *SAINT*; program(s) used to solve structure: *SHELXS97* (Sheldrick, 2008 ▶); program(s) used to refine structure: *SHELXL97* (Sheldrick, 2008 ▶); molecular graphics: *SHELXTL* (Sheldrick, 2008 ▶); software used to prepare material for publication: *SHELXTL*.

## Supplementary Material

Crystal structure: contains datablock(s) I, global. DOI: 10.1107/S1600536813031826/lh5669sup1.cif

Structure factors: contains datablock(s) I. DOI: 10.1107/S1600536813031826/lh5669Isup2.hkl

Additional supplementary materials:  crystallographic information; 3D view; checkCIF report

## Figures and Tables

**Table 1:** Hydrogen-bond geometry (Å, °)

*D*—H⋯*A*	*D*—H	H⋯*A*	*D*⋯*A*	*D*—H⋯*A*
C5—H5⋯O2^i^	0.93	2.54	3.388 (6)	152

Symmetry code: (i) 
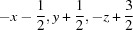
.

**Fig. 1.**
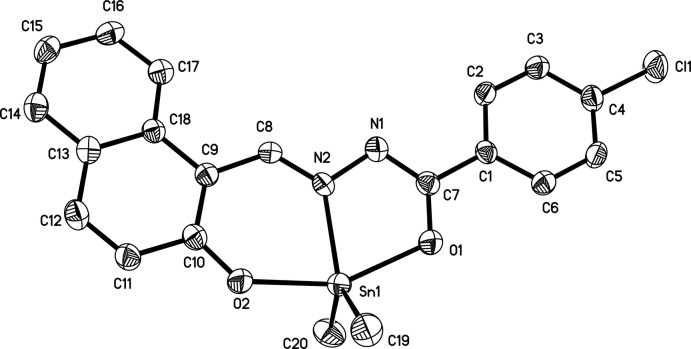
The molecular structure of the title compound, showing 50% probability displacement ellipsoids. H atoms have been omitted for clarity.

## supplementary crystallographic information

### 1. Comment

The chemistry of organotin(IV) derivatives is a subject of study with growing interest due to their significant antimicrobial properties as well as antitumor activities (Hong *et al.*, 2013). As a part of our ongoing investigations in this field we have synthesized the title compound and present its crystal structure here. The molecular structure of the title compound, (I), is shown in Fig. 1. The Sn atom has distorted trigonal-bipyramidal geometry, with atoms O1 and O2 in axial positions [O1—Sn1—O2 = 153.39 (13) °] and the atoms C19, C20 and N2 in equatorial positions. The sum of the equatorial angles is 359.9 °, indicating approximate coplanarity for these atoms. The Sn1—N2 bond length is 2.160 (4) Å close to the sum of the non-polar covalent radii 2.15 Å, indicating a strong Sn—N interaction. The O atoms coordinate to the Sn atom with one shorter [2.092 (3) Å] and one longer [2.144 (3) Å] bond. The dihedral angle between the naphthalene ring system (C9-C18) and the benzene ring (C1-C6) is 8.6 (2)°.

### 2. Experimental

2-hydroxy-1-naphthaldehyde 4-chlorobenzoylhydrazone (1 mmol) and sodium ethoxide (1 mmol) were added to the solution of dry methanol(30 ml) and stirred for 10 mins. Dimeyltin dichloride (1 mmol) was then added to the reactor and the reaction mixture was stirred for 4 h. The resulting clear solution was evaporated under vacuum. The product was crystallized from a mixture of dichloromethane/ethanol(1:1) to yield orange blocks of the title compound (yield 73%).

### 3. Refinement

The H atoms were positioned geometrically, with methyl C—H distances of 0.96 Å and aromatic and aldehydic C—H distances of 0.93 Å, and refined as riding on their parent atoms, with *U*_iso_(H) = 1.2 *U*_eq_(C) or 1.5 *U*_eq_(C) for the methyl groups.

### Crystal data

**Table:**

[Sn(CH_3_)_2_(C_18_H_11_ClN_2_O_2_)]	*F*(000) = 936
*M**_r_* = 471.50	*D*_x_ = 1.655 Mg m^−^^3^
Monoclinic, *P*2_1_/*n*	Mo *K*α radiation, λ = 0.71073 Å
Hall symbol: -P 2yn	Cell parameters from 2600 reflections
*a* = 8.7927 (8) Å	θ = 2.6–28.5°
*b* = 17.4170 (15) Å	µ = 1.51 mm^−^^1^
*c* = 12.5014 (12) Å	*T* = 293 K
β = 98.690 (9)°	Block, orange
*V* = 1892.5 (3) Å^3^	0.24 × 0.23 × 0.13 mm
*Z* = 4	

### Data collection

**Table:**

Siemens SMART CCD area-detector diffractometer	3343 independent reflections
Radiation source: fine-focus sealed tube	2584 reflections with *I* > 2σ(*I*)
Graphite monochromator	*R*_int_ = 0.047
φ and ω scans	θ_max_ = 25.0°, θ_min_ = 2.6°
Absorption correction: multi-scan (*SADABS*; Sheldrick, 1996)	*h* = −8→10
*T*_min_ = 0.714, *T*_max_ = 0.828	*k* = −20→19
11127 measured reflections	*l* = −14→14

### Refinement

**Table:**

Refinement on *F*^2^	Primary atom site location: structure-invariant direct methods
Least-squares matrix: full	Secondary atom site location: difference Fourier map
*R*[*F*^2^ > 2σ(*F*^2^)] = 0.040	Hydrogen site location: inferred from neighbouring sites
*wR*(*F*^2^) = 0.107	H-atom parameters constrained
*S* = 1.06	*w* = 1/[σ^2^(*F*_o_^2^) + (0.0488*P*)^2^ + 0.4409*P*] where *P* = (*F*_o_^2^ + 2*F*_c_^2^)/3
3343 reflections	(Δ/σ)_max_ = 0.001
237 parameters	Δρ_max_ = 0.83 e Å^−^^3^
0 restraints	Δρ_min_ = −0.61 e Å^−^^3^

### Fractional atomic coordinates and isotropic or equivalent isotropic displacement parameters (Å^2^)

**Table:**

	*x*	*y*	*z*	*U*_iso_*/*U*_eq_	
Sn1	−0.14514 (4)	0.026153 (19)	0.73069 (3)	0.04369 (15)	
O1	−0.1839 (4)	0.1313 (2)	0.8117 (3)	0.0542 (9)	
O2	−0.0073 (4)	−0.0641 (2)	0.6906 (3)	0.0597 (10)	
N1	0.0353 (5)	0.1015 (2)	0.9316 (3)	0.0511 (10)	
N2	0.0421 (5)	0.0380 (2)	0.8630 (3)	0.0447 (10)	
C1	−0.1055 (5)	0.2148 (3)	0.9621 (4)	0.0408 (11)	
C2	−0.0132 (5)	0.2290 (3)	1.0615 (4)	0.0457 (12)	
H2	0.0645	0.1945	1.0874	0.055*	
C3	−0.0355 (6)	0.2931 (3)	1.1216 (4)	0.0532 (13)	
H3	0.0261	0.3019	1.1878	0.064*	
C4	−0.1512 (6)	0.3443 (3)	1.0820 (4)	0.0528 (13)	
C5	−0.2422 (6)	0.3321 (3)	0.9852 (4)	0.0534 (13)	
H5	−0.3192	0.3671	0.9599	0.064*	
C6	−0.2204 (6)	0.2679 (3)	0.9248 (4)	0.0503 (12)	
H6	−0.2826	0.2599	0.8587	0.060*	
C7	−0.0849 (5)	0.1448 (3)	0.8972 (4)	0.0446 (11)	
C8	0.1604 (5)	−0.0072 (3)	0.8915 (4)	0.0420 (11)	
H8	0.2257	0.0062	0.9543	0.050*	
C9	0.2008 (5)	−0.0748 (3)	0.8370 (4)	0.0411 (11)	
C10	0.1179 (6)	−0.0978 (3)	0.7378 (4)	0.0466 (12)	
C11	0.1701 (6)	−0.1620 (3)	0.6826 (4)	0.0530 (13)	
H11	0.1140	−0.1776	0.6170	0.064*	
C12	0.2989 (6)	−0.2008 (3)	0.7233 (4)	0.0555 (13)	
H12	0.3304	−0.2420	0.6847	0.067*	
C13	0.3880 (5)	−0.1797 (3)	0.8248 (4)	0.0454 (12)	
C14	0.5222 (6)	−0.2202 (3)	0.8669 (4)	0.0543 (13)	
H14	0.5554	−0.2602	0.8268	0.065*	
C15	0.6041 (6)	−0.2024 (3)	0.9642 (4)	0.0551 (13)	
H15	0.6932	−0.2294	0.9904	0.066*	
C16	0.5533 (6)	−0.1430 (3)	1.0250 (4)	0.0539 (13)	
H16	0.6072	−0.1315	1.0930	0.065*	
C17	0.4242 (5)	−0.1012 (3)	0.9852 (4)	0.0478 (12)	
H17	0.3932	−0.0614	1.0267	0.057*	
C18	0.3378 (5)	−0.1170 (3)	0.8833 (4)	0.0413 (11)	
C19	−0.1543 (7)	0.0779 (4)	0.5774 (4)	0.0695 (16)	
H19A	−0.0524	0.0920	0.5662	0.104*	
H19B	−0.2178	0.1229	0.5739	0.104*	
H19C	−0.1970	0.0423	0.5223	0.104*	
C20	−0.3234 (7)	−0.0415 (4)	0.7770 (5)	0.0732 (18)	
H20A	−0.3626	−0.0755	0.7189	0.110*	
H20B	−0.4047	−0.0088	0.7932	0.110*	
H20C	−0.2841	−0.0711	0.8399	0.110*	
Cl1	−0.1839 (2)	0.42381 (10)	1.16029 (14)	0.0880 (5)	

### Atomic displacement parameters (Å^2^)

**Table:**

	*U*^11^	*U*^22^	*U*^33^	*U*^12^	*U*^13^	*U*^23^
Sn1	0.0436 (2)	0.0429 (2)	0.0436 (2)	−0.00084 (15)	0.00346 (16)	−0.00068 (16)
O1	0.0518 (19)	0.054 (2)	0.054 (2)	0.0098 (17)	0.0001 (17)	−0.0102 (18)
O2	0.057 (2)	0.067 (2)	0.050 (2)	0.017 (2)	−0.0076 (17)	−0.0145 (19)
N1	0.048 (2)	0.047 (3)	0.059 (3)	0.004 (2)	0.006 (2)	−0.007 (2)
N2	0.048 (2)	0.039 (2)	0.046 (2)	0.0002 (18)	0.0058 (19)	−0.0097 (19)
C1	0.042 (2)	0.034 (3)	0.048 (3)	−0.004 (2)	0.010 (2)	0.000 (2)
C2	0.040 (3)	0.044 (3)	0.053 (3)	0.005 (2)	0.006 (2)	0.002 (2)
C3	0.051 (3)	0.056 (3)	0.050 (3)	0.006 (3)	−0.001 (2)	−0.006 (3)
C4	0.060 (3)	0.044 (3)	0.055 (3)	0.007 (3)	0.010 (3)	−0.005 (3)
C5	0.057 (3)	0.049 (3)	0.051 (3)	0.018 (3)	0.000 (3)	0.001 (3)
C6	0.052 (3)	0.050 (3)	0.046 (3)	0.005 (2)	0.000 (2)	0.001 (3)
C7	0.045 (3)	0.040 (3)	0.048 (3)	−0.004 (2)	0.008 (2)	0.000 (2)
C8	0.040 (3)	0.044 (3)	0.040 (3)	−0.001 (2)	0.001 (2)	−0.003 (2)
C9	0.040 (2)	0.041 (3)	0.043 (3)	−0.002 (2)	0.009 (2)	−0.001 (2)
C10	0.050 (3)	0.046 (3)	0.045 (3)	0.000 (2)	0.011 (2)	−0.004 (2)
C11	0.064 (3)	0.052 (3)	0.041 (3)	0.009 (3)	0.002 (2)	−0.005 (3)
C12	0.066 (3)	0.050 (3)	0.052 (3)	0.008 (3)	0.015 (3)	−0.006 (3)
C13	0.049 (3)	0.043 (3)	0.047 (3)	0.002 (2)	0.015 (2)	0.005 (2)
C14	0.058 (3)	0.045 (3)	0.062 (3)	0.007 (3)	0.013 (3)	0.004 (3)
C15	0.048 (3)	0.052 (3)	0.066 (3)	0.007 (2)	0.007 (3)	0.014 (3)
C16	0.044 (3)	0.060 (3)	0.055 (3)	−0.003 (3)	−0.002 (2)	0.009 (3)
C17	0.050 (3)	0.046 (3)	0.048 (3)	−0.003 (2)	0.009 (2)	0.005 (2)
C18	0.044 (3)	0.037 (3)	0.044 (3)	−0.003 (2)	0.008 (2)	0.002 (2)
C19	0.091 (4)	0.066 (4)	0.051 (3)	0.007 (3)	0.008 (3)	0.008 (3)
C20	0.067 (4)	0.073 (4)	0.082 (5)	−0.025 (3)	0.018 (4)	−0.008 (3)
Cl1	0.1110 (13)	0.0687 (10)	0.0780 (10)	0.0321 (9)	−0.0065 (9)	−0.0259 (9)

### Geometric parameters (Å, º)

**Table:**

Sn1—O2	2.092 (3)	C9—C10	1.399 (7)
Sn1—C19	2.108 (5)	C9—C18	1.455 (6)
Sn1—C20	2.111 (5)	C10—C11	1.426 (7)
Sn1—O1	2.144 (3)	C11—C12	1.351 (7)
Sn1—N2	2.160 (4)	C11—H11	0.9300
O1—C7	1.294 (5)	C12—C13	1.435 (7)
O2—C10	1.306 (6)	C12—H12	0.9300
N1—C7	1.317 (6)	C13—C14	1.406 (7)
N1—N2	1.407 (5)	C13—C18	1.422 (6)
N2—C8	1.310 (6)	C14—C15	1.353 (7)
C1—C6	1.397 (7)	C14—H14	0.9300
C1—C2	1.400 (7)	C15—C16	1.397 (7)
C1—C7	1.489 (6)	C15—H15	0.9300
C2—C3	1.377 (7)	C16—C17	1.377 (7)
C2—H2	0.9300	C16—H16	0.9300
C3—C4	1.387 (7)	C17—C18	1.408 (6)
C3—H3	0.9300	C17—H17	0.9300
C4—C5	1.362 (7)	C19—H19A	0.9600
C4—Cl1	1.745 (5)	C19—H19B	0.9600
C5—C6	1.379 (7)	C19—H19C	0.9600
C5—H5	0.9300	C20—H20A	0.9600
C6—H6	0.9300	C20—H20B	0.9600
C8—C9	1.431 (6)	C20—H20C	0.9600
C8—H8	0.9300		
			
O2—Sn1—C19	92.74 (19)	C10—C9—C18	119.8 (4)
O2—Sn1—C20	97.3 (2)	C8—C9—C18	118.5 (4)
C19—Sn1—C20	124.0 (3)	O2—C10—C9	124.2 (4)
O2—Sn1—O1	153.39 (13)	O2—C10—C11	116.4 (4)
C19—Sn1—O1	94.45 (19)	C9—C10—C11	119.4 (5)
C20—Sn1—O1	99.8 (2)	C12—C11—C10	121.5 (5)
O2—Sn1—N2	81.53 (13)	C12—C11—H11	119.2
C19—Sn1—N2	125.1 (2)	C10—C11—H11	119.2
C20—Sn1—N2	110.8 (2)	C11—C12—C13	121.4 (5)
O1—Sn1—N2	73.39 (13)	C11—C12—H12	119.3
C7—O1—Sn1	114.3 (3)	C13—C12—H12	119.3
C10—O2—Sn1	135.1 (3)	C14—C13—C18	120.1 (5)
C7—N1—N2	111.0 (4)	C14—C13—C12	121.2 (5)
C8—N2—N1	114.3 (4)	C18—C13—C12	118.8 (4)
C8—N2—Sn1	129.3 (3)	C15—C14—C13	121.6 (5)
N1—N2—Sn1	116.3 (3)	C15—C14—H14	119.2
C6—C1—C2	118.2 (4)	C13—C14—H14	119.2
C6—C1—C7	120.1 (4)	C14—C15—C16	119.3 (5)
C2—C1—C7	121.7 (4)	C14—C15—H15	120.4
C3—C2—C1	121.1 (4)	C16—C15—H15	120.4
C3—C2—H2	119.4	C17—C16—C15	120.6 (5)
C1—C2—H2	119.4	C17—C16—H16	119.7
C2—C3—C4	118.9 (5)	C15—C16—H16	119.7
C2—C3—H3	120.5	C16—C17—C18	121.7 (5)
C4—C3—H3	120.5	C16—C17—H17	119.1
C5—C4—C3	121.2 (5)	C18—C17—H17	119.1
C5—C4—Cl1	119.8 (4)	C17—C18—C13	116.7 (4)
C3—C4—Cl1	118.9 (4)	C17—C18—C9	124.2 (4)
C4—C5—C6	120.1 (5)	C13—C18—C9	119.1 (4)
C4—C5—H5	120.0	Sn1—C19—H19A	109.5
C6—C5—H5	120.0	Sn1—C19—H19B	109.5
C5—C6—C1	120.5 (5)	H19A—C19—H19B	109.5
C5—C6—H6	119.7	Sn1—C19—H19C	109.5
C1—C6—H6	119.7	H19A—C19—H19C	109.5
O1—C7—N1	124.8 (5)	H19B—C19—H19C	109.5
O1—C7—C1	118.5 (4)	Sn1—C20—H20A	109.5
N1—C7—C1	116.7 (4)	Sn1—C20—H20B	109.5
N2—C8—C9	127.5 (4)	H20A—C20—H20B	109.5
N2—C8—H8	116.3	Sn1—C20—H20C	109.5
C9—C8—H8	116.3	H20A—C20—H20C	109.5
C10—C9—C8	121.6 (4)	H20B—C20—H20C	109.5

### Hydrogen-bond geometry (Å, º)

**Table:**

*D*—H···*A*	*D*—H	H···*A*	*D*···*A*	*D*—H···*A*
C5—H5···O2^i^	0.93	2.54	3.388 (6)	152

Symmetry code: (i) −*x*−1/2, *y*+1/2, −*z*+3/2.
